# A Retrospective Cohort Study of the Effects of Canal Filling Ratio and Femoral Bone Density Change on the Outcomes of Anatomical and Double-tapered Wedge Stems

**DOI:** 10.1055/s-0044-1787770

**Published:** 2024-08-01

**Authors:** Thakrit Chompoosang, Patcharavit Ploynumpon

**Affiliations:** 1 Departamento de Ortopedia, Faculdade de Medicina, Rajavithi Hospital, Bangkok, Tailândia

**Keywords:** bone remodeling, femur, hip prosthesis, prosthesis design

## Abstract

**Objective** This study aims to compare the proximal femoral bone density changes in follow-up X-ray imaging and the proximal filling ratios of stems between anatomical and double-tapered wedge stem designs.

**Methods** Patients aged between 18 and 80 years who received primary total hip arthroplasty using both types of stems between 2017 and 2019 and had follow-up tests for up to a year were included in the study. Canal filling ratios at 3 levels (lesser trochanter [LT], 2 cm above LT, and 7 cm below LT) using the optimal densitometry method. Femoral bone density changes were measured using the Gruen zoning method.

**Results** A total of 92 patients (76% female and 24% male) met the inclusion criteria for this study. The mean age was 53.86 ± 13.00 years. The canal filling ratio in the double-tapered wedge group (Accolade II) was significantly higher than that in the anatomical stem group (ABGII) ( *p* < 0.001, *p* < 0.001, and *p* = 0.013) for all levels of measurement. No significant difference was observed between both types of stems in femoral bone density changes in zones 1 and 4. However, there were significant differences in femoral bone change, with bone loss being higher in the anatomical stem group in zone 7 (−25% versus −17%; *p* = 0.010).

**Conclusion** Double-tapered wedge stem had a significantly higher canal filling ratio than the anatomical stem at all levels but had less femoral bone density loss in the follow-up postoperative imaging in zone 7. Furthermore, in zones 1 and 4, there was no significant difference in femoral bone density loss.

## Introduction

Total hip arthroplasty is one of the most common orthopedic surgical procedures. As the number of surgeries increased, the stem design became one of the most important factors affecting overall prosthesis longevity and patient satisfaction. One of the stems that provide good results and long-term outcomes is the cementless one, invented in 1950. 1 Several studies have reported early loosening and instability associated with the initial design, which may be caused by proximal femoral osteopenia resulting from the stress shielding effect. 2 Many modern stems have been developed by promoting proximal engagement, using hydroxyapatite porous coating, which is more compatible with the patient proximal femoral dense bone. By utilizing taper and anatomical designs, they can decrease the distal stem engagement and employ shorter stems, which can reduce proximal bone loss by up to 14%. 3 Moreover, many studies have shown that the stem design revolution reduced stem subsidence, thigh pain, and loosening. 4 5 Nevertheless, no previous research has compared the progression of bone integration and proximal bone loss between a double-tapered wedge stems (Accolade II, Stryker, Portage, MI, USA) and anatomical stems (ABGII, Stryker). Therefore, this study compares proximal femoral filling differences between such stem designs, using immediate postoperative imaging and proximal bone loss utilizing follow-up X-ray. The results will provide a better choice of stem, decrease early complications, and increase satisfaction with the total hip replacement operation.

## Materials and Methods

### Study Design

This study is a retrospective descriptive-cohort study of immediate and postoperative follow-up imaging from total hip arthroplasty surgery performed from 2017 to 2019. The hospital's Ethics Committee (ID: 62133) approved the research protocol and waived the requirement for informed consent for this study. All patients' collected data and identifiers were made fully anonymous.

## Samples

### Inclusion Criteria

With permission from the radiology department of Rajavithi, patients aged between 18 and 80 years who received primary total hip arthroplasty using both types of stems between 2017 and 2019 and had follow-up imaging for up to a year were included in this study.

### Exclusion Criteria

Patients who were under 18 years of age, received revision hip arthroplasty, had prior hip dysplasia, had any postoperative complication, and with follow-up imaging of less than 1 year were excluded.

### Data Collection and Measurement

**Fig. 1:**
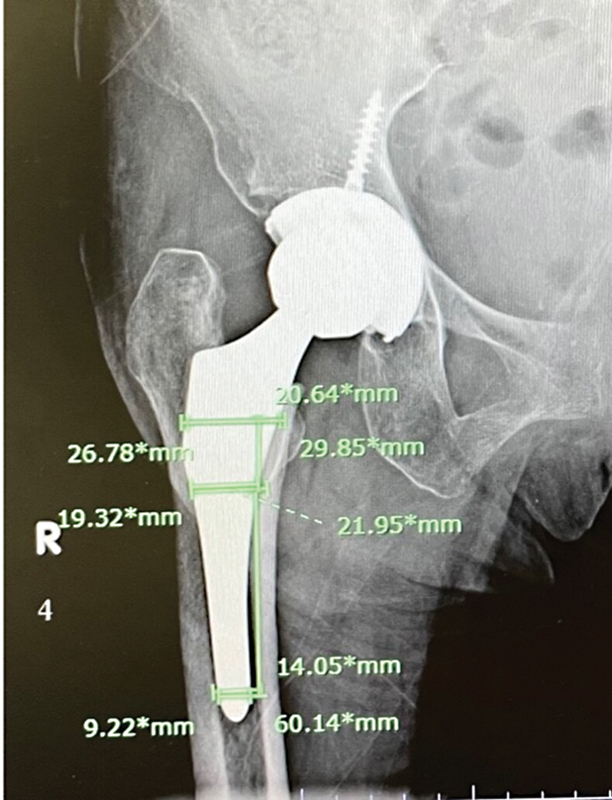
Measurement of canal filling ratios at the lesser trochanter (LT), 2 cm proximal to LT, and 6 cm distal to LT.

We obtained immediate and postoperative follow-up imaging from the radiology department for patients who underwent total hip arthroplasty, covering a period of up to 1 year. Data analysis was based on the femoral canal filling ratio method, used by the orthopedic surgeon responsible for adult hip and knee reconstruction. 6 The data included measurements of the proximal femoral and stem diameter in the anteroposterior view at three levels: lesser trochanter (LT), 2 cm proximal to the LT, and 6 cm distal to the LT ( Fig. 1 ).

**Fig. 2:**
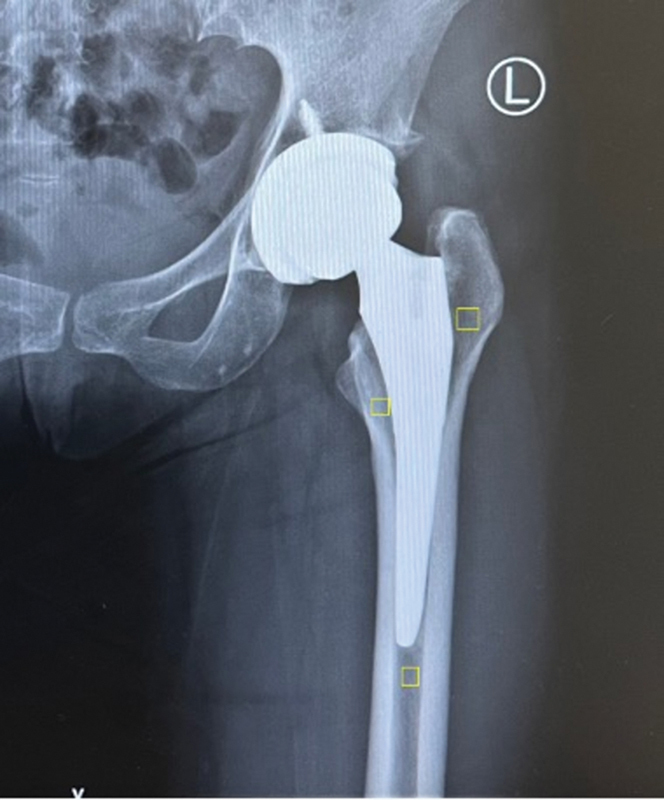
Measurement of the proximal femoral bone density using optimal densitometry method.

The follow-up imaging was analyzed for proximal femoral bone density changes using the optimal densitometry method, 7 employing Image J (public domain), a digital optical image analysis software for windows, which measured bone changes in zones 1, 4, and 7 according to the Gruen fixation zone 8 ( Fig. 2 ).

### Statical Analysis

Descriptive statistics (number, percentage, mean, median, standard deviation, as well as minimum and maximum values) were used to describe the characteristics of the samples. The Chi-squared test was employed to compare categorical data. The paired *t* test was utilized to compare independent data such as stem and femoral types. Furthermore, the *t* test was employed to compare dependent data such as postoperative imaging. The level of significance was defined as a *p* -value < 0.05. All statistical analyses were performed using the IBM SPSS Statistics for Windows (IBM Corp., Armonk, NY, USA) software, version 20.0.

## Results

### Demographics Data

A total sample of 92 patients (22 males and 70 females) was included in this study. The mean age was of 53.86 ± 13.00 years. There were 34 patients in the anatomical stem group (ABGII) and 58 patients in the double-tapered wedge stem group (Accolade II).

When comparing canal filling ratios between both stems, the canal filling ratio in the double-tapered wedge stem was significantly higher than that in the anatomical stem group at all 3 measurement levels ( *p* < 0.001, *p* < 0.001, and *p* = 0.013), as shown in Table 1 .

**Table 1:** Comparison of canal filling ratios between two types of stems

Level	Anatomical stem (n = 34)	Double-tapered wedge stem (n = 58)	Difference (95% confidence interval)	*p* -value
Lesser trochanter	81.56	88.13	−6.57 (−9.74 to −3.39)	< 0.001*
2 cm above the lesser trochanter	85.98	93.49	−7.51 (−10.07 to −4.95)	< 0.001*
6 cm below the lesser trochanter	78.58	85.64	−7.06 (−12.56 to −1.56)	0.013*

Note: *Statistical significant ( *p* -value <0.05).

Table 2 presents the femoral bone density changes in each stem type in Gruen zones 1, 4, and 7. Both stems showed a femoral proximal bone loss from the baseline to every time point.

1. Proximal bone density changes in anatomical stem
At 6-months postoperatively, there were significant differences in femoral bone loss in zones 1, 4, and 7 ( *p* = 0.024, *p* < 0.001, and *p* = 0.006, respectively); the highest femoral bone loss was observed in zone 4 (5.74%). At 1-year postoperatively, there was a significant difference in femoral bone loss in zones 1, 4, and 7 ( *p* < 0.001); the highest femoral bone loss was found in zone 7 (20.65%). At 2 years postoperatively, significant differences in femoral bone loss were observed in zones 1 ( *p* < 0.001), 4 ( *p* = 0.004), and 7 ( *p* < 0.001); the highest femoral bone loss was seen in zone 1 (34.48%).
2. Proximal bone density changes in double-tapered wedge stem
At 6-months postoperatively, there was a significant difference in femoral bone loss in zones 1, 4, and 7 ( *p* < 0.001); the highest femoral bone loss was observed in zone 1 (8.28%). At 1-year postoperatively, there was a significant difference in femoral bone loss in zones 1, 4, and 7 ( *p* < 0.001); the highest femoral bone loss was seen in zone 1 (12.48%). At 2-years postoperatively, a significant difference in the femoral bone loss was found in zones 1, 4, and 7 ( *p* < 0.001); the highest femoral bone loss was seen in zone 1 (22.37%).

**Table 2:** Femoral bone density changes in each stem type in Gruen zones 1, 4, and 7

Femur	Anatomical stem (n = 34)				Double-tapered wedge stem (n = 58)			
	Postoperative (baseline)	6 months	1 year	2 years	Postoperative (baseline)	6 months	1 year	2 years
**Zone 1** FBD (%; mean ± SD) Change (%)	(135.59 ± 12.20)	(131.15 ± 10.53) −4.44	(117.35 ± 14.19) −13.79	(84.74 ± 7.29) −34.48	(130.33 ± 13.30)	(122.05 ± 15.14) −8.28	(109.57 ± 12.09) −12.48	(91.63 ± 9.06) −22.37
*p* -value		0.024*	< 0.001*	< 0.001*		< 0.001*	< 0.001*	< 0.001*
**Zone 4** FBD (%; mean ± SD) Change (%)	(164.03 ± 16.41)	(158.29 ± 17.11) −5.74	(145.74 ± 16.92) −12.56	(135.39 ± 12.74) −13.65	(152.86 ± 29.44)	(148.14 ± 24.19) −4.72	(138.64 ± 18.49) −9.50	(129.77 ± 11.34) −16.23
*p* -value		0.001*	< 0.001*	0.004*		0.098	< 0.001*	< 0.001*
**Zone 7** FBD (%; mean ± SD) Change (%)	(157.85 ± 13.84)	(152.71 ± 11.31) −5.15	(132.06 ± 12.33) −20.65	(107.83 ± 19.79) −29.57	(152.29 ± 13.11)	(144.29 ± 14.85) −8.00	(135.12 ± 14.89) −9.17	(126.87 ± 10.59) −11.13
*p* -value		0.006	< 0.001*	< 0.001*		< 0.001*	< 0.001*	< 0.001*

**Abbreviations:** FBD, femoral bone density; SD, standard deviation.

Note: *Statistical significant ( *p* -value <0.05).

**Fig. 3:**
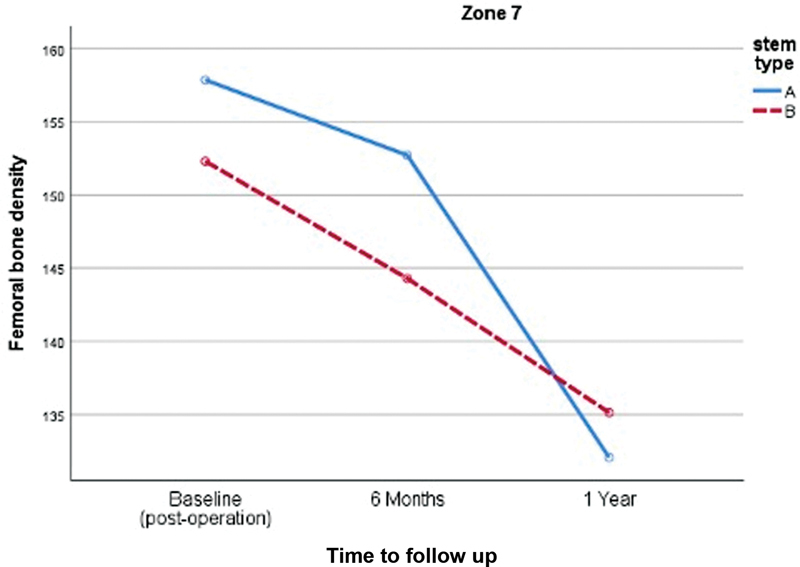
Comparison of the proximal femoral bone density changes in the Gruen zone 7 of both stems (A, anatomical stem; B, double-tapered wedge stem).

**Fig. 4:**
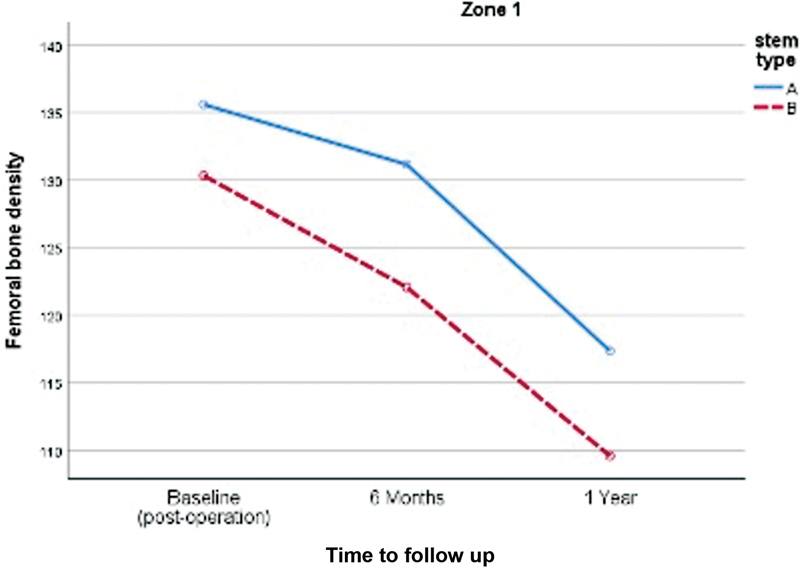
Comparison of the proximal femoral bone density changes in the Gruen zone 1 of both stems (A, anatomical stem; B, double-tapered wedge stem).

Comparing proximal femoral bone loss between both designs, the double-tapered wedge stem demonstrated significantly less proximal femoral bone loss in the Gruen zone 7 ( Fig. 3 ). However, there was no significant difference in proximal femoral bone loss in zones 1 and 4, as shown in ( Figs. 4 5 ) and Table 3 .

**Fig. 5:**
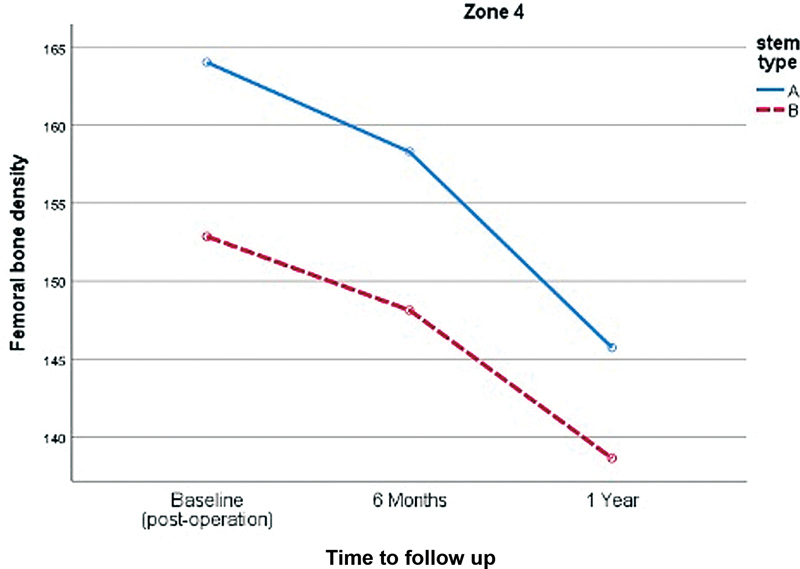
Comparison of the proximal femoral bone density changes in the Gruen zone 4 of both stems (A, anatomical stem; B, double-tapered wedge stem).

**Table 3:** Comparison of femoral bone density changes in each zone in both types of stems

Femoral bone density	Stem type		*p* -value
	Anatomical stem (n = 34)	Double-tapered wedge stem (n = 58)	
**Zone 1**			
Postoperative (baseline)	135.59 ± 12.20	130.33 ± 13.30	0.062
6 months	131.15 ± 10.53	122.05 ± 15.14	0.003*
1 year	117.35 ± 14.19	109.57 ± 12.09	0.006*
2 years	84.74 ± 7.29	91.63 ± 9.06	0.004*
Change (1-year postoperatively)	−18.24 ± 20.48	−20.76 ± 9.36	0.501
**Zone 4**			
Postoperative (baseline)	164.03 ± 16.41	152.86 ± 29.44	0.022*
6 months	158.29 ± 17.11	148.14 ± 24.19	0.021*
1 year	145.74 ± 16.92	138.64 ± 18.49	0.070
2 years	135.39 ± 12.74	129.77 ± 11.34	0.096
Change (1-year postoperatively)	−18.29 ± 14.79	−14.22 ± 24.22	0.320
**Zone 7**			
Postoperative (baseline)	157.85 ± 13.84	152.29 ± 13.11	0.058
6 months	152.71 ± 11.31	144.29 ± 14.85	0.005*
1 year	132.06 ± 12.33	135.12 ± 14.89	0.314
2 years	107.83 ± 19.79	126.87 ± 10.59	< 0.001*
Change (1-year postoperatively)	−25.79 ± 15.85	−17.17 ± 13.23	0.010*

Note: *Statistical significant ( *p* -value <0.05).

Table 3 showed that only zone 7 had significant differences in femoral bone density changes between both stems ( *p* = 0.01).

## Discussion

Cementless total hip arthroplasty is a popular procedure, particularly for younger patients, 9 with a good long-term outcome. However, it was reported to have proximal femoral osteopenia and early aseptic loosening in early designs 4 due to the stress shielding effect and proximal micromotion of the stem. Later, the stem was refined by improvement in surface and coating material, decrease in the material's stiffness, and variants of the femoral stem length all greatly improve the survival and lessens chance of complications in the procedure. 10

For canal filling of femoral canal, our study found the canal filling ratio in the double-tapered wedge stem was significantly higher than that in the anatomical stem group at all levels (LT, 2 cm above LT, and 6 cm below LT). It is worth noting that the higher femoral canal filling and canal filling ratio observed in our study may increase the risk of failure of osteointegration, as suggested by the study by Cooper et al., 11 who noted that an increase in the mid and distal filling, as well as and canal-flare index, are the most important risk factors for osteointegration failure.

According to our study on changes in periprosthetic bone density, the immediate postoperative bone mineral density on the operated side should be used as the baseline value to exclude bone loss due to the operation procedure. 12 Despite this method, our study found bone density loss in both stems from the baseline, which we attribute to stress-shielding in the area. This finding is consistent with the Venesmaa et al. 13 study, which reported a general decrease in all regions of interest until 6 months, particularly in the Calcar region, and only minor changes after this time period. However, our study observed a decrease in bone density up to 1 year postoperatively. We found that in zone 7, the anatomical stem had a significantly higher femoral bone density loss than that the double-tapered wedge stem (−25% versus −17%, *p* = 0.010). Nevertheless, no significant femoral bone density loss was observed in zones 1 and 4.

The main strength of this study is that it was conducted by a single surgeon in a single center, which minimized confounding factors from surgical technique and postoperative patient care. However, this study has limitations. First, its retrospective nature, which limited data collection on all factors that may have influenced bone loss in our patient population. Additionally, the sample size was relatively small, which prevented us from conducting a meaningful subgroup analysis to investigate the impact of various factors on bone loss. Second, the follow-up period was short (12 months), although we believe it was adequate, as periprosthetic bone density loss was most pronounced in the first postoperative year, with minimal changes thereafter. This finding is consistent with previous studies that highlighted the initial periprosthetic bone remodeling process occurring within the first 12 postoperative months. 14

## Conclusion

Double-tapered wedge stem design had a significantly higher canal filling ratio than the anatomical stem at all levels, with a lower femoral bone density loss identified in the follow-up postoperative imaging at zone 7. However, in zones 1 and 4, no significant difference in femoral bone density loss was observed.

## Supplementary Material

Supporting Information Supporting Information

Informações de Apoio Informações de Apoio
