# Supplementary material for: A Retrospective Cohort Study of the Effects of Canal Filling Ratio and Femoral Bone Density Change on the Outcomes of Anatomical and Double-tapered Wedge Stems
Source: Rev Bras Ortop (Sao Paulo). 2024 Aug 1;59(5):e752–7. doi: 10.1055/s-0044-1787770 (PMC11624936; doi:10.1055/s-0044-1787770)
Supplement: Supplementary file 1 — Supporting Information [file 10-1055-s-0044-1787770-s2400011en.pdf]

Supporting Information

Table S1 Raw dataset of all measurements in the present study

Ploynumpon P, Chompoosang T. Comparison Canal Filling Ratio And Femoral Bone Density Change Between Wedge Taper And Anatomical Stem Design. medRxiv [Internet]. 2022; Available from: https://www.medrxiv.org/content/early/2022/11/17/2022.11.08.22282094. **Doi:** 10.1101/2022.11.08.22282094.

 © 2024. The Author(s).
