# Supplementary material for: A Retrospective Cohort Study of the Effects of Canal Filling Ratio and Femoral Bone Density Change on the Outcomes of Anatomical and Double-tapered Wedge Stems
Source: Rev Bras Ortop (Sao Paulo). 2024 Aug 1;59(5):e752–7. doi: 10.1055/s-0044-1787770 (PMC11624936; doi:10.1055/s-0044-1787770)
Supplement: Supplementary file 2 — Informações de Apoio [file 10-1055-s-0044-1787770-s2400011pt.pdf]

## Informações de Apoio

**Tabela S1** Conjunto de dados crus de todas as medidas deste estudo

Ploynumpon P, Chompoosang T. Comparison Canal Filling Ratio And Femoral Bone Density Change Between Wedge Taper And Anatomical Stem Design. medRxiv [Internet]. 2022; Disponível em: https://www.medrxiv.org/content/early/2022/11/17/2022.11.08.22282094 **Doi:** 10.1101/2022.11.08.22282094.

 © 2024. The Author(s).
