# Supplementary material for: A Retrospective Cohort Study of the Effects of Canal Filling Ratio and Femoral Bone Density Change on the Outcomes of Anatomical and Double-tapered Wedge Stems
Source: Rev Bras Ortop (Sao Paulo). 2024 Aug 1;59(5):e752–7. doi: 10.1055/s-0044-1787770 (PMC11624936; doi:10.1055/s-0044-1787770)
Supplement: Related texts

# Research article: Um estudo de coorte retrospectiva dos efeitos da taxa de preenchimento do canal e da alteração da densidade óssea femoral nos desfechos de hastes anatômicas e cônicas de cunha dupla

**Keywords:** desenho de prótese, fêmur, prótese de quadril, remodelação óssea

## Abstract

**Objetivo** Este estudo teve como objetivo comparar as alterações na densidade óssea femoral proximal em radiografias de acompanhamento e as taxas de preenchimento proximal das hastes anatômicas ou cônicas de cunha dupla.

**Métodos** O estudo incluiu pacientes com idade entre 18 e 80 anos submetidos à artroplastia total primária do quadril com os dois tipos de hastes entre 2017 e 2019 e que tinham radiografias de acompanhamento por até um ano. As taxas de preenchimento do canal em três níveis (trocânter menor [TM], 2 cm acima do TM e 7 cm abaixo do TM) foram determinadas pelo método de densitometria ideal. As alterações na densidade óssea femoral foram medidas pelo método de zoneamento de Gruen.

**Resultados** Ao todo, 92 pacientes (76% do sexo feminino e 24% do sexo masculino) atenderam aos critérios de inclusão deste estudo. A média de idade foi de 53,86 ± 13,00 anos. A taxa de preenchimento do canal no grupo com hastes cônicas de cunha dupla (Accolade II) foi significativamente maior do que no grupo com haste anatômica (ABGII) ( *p* < 0,001, *p* < 0,001 e *p* = 0,013) em todos os níveis de medição. Não foi notada diferença significativa entre os dois tipos de hastes nas alterações da densidade óssea femoral nas zonas 1 e 4. Porém, diferenças significativas na alteração óssea femoral foram detectadas, já que a perda óssea foi maior no grupo com haste anatômica, na zona 7 (−25% *versus* −17%; *p* = 0,010).

**Conclusão** A haste cônica de cunha dupla gerou uma taxa de preenchimento do canal significativamente maior do que a haste anatômica em todos os níveis, mas com menor perda de densidade óssea femoral em radiografias pós-operatórias de acompanhamento na zona 7. Além disso, nas zonas 1 e 4, não houve perda significativa de densidade óssea femoral.

## Introdução

A artroplastia total do quadril é um dos procedimentos cirúrgicos ortopédicos mais comuns. À medida que o número de cirurgias aumentou, o design da haste tornou-se um dos fatores mais importantes que afetam a longevidade geral da prótese e a satisfação do paciente. Um dos modelos de haste que proporcionam bons resultados e desfechos em longo prazo é a haste não cimentada, inventada em 1950. 1 Vários estudos relataram afrouxamento precoce e instabilidade associada ao design inicial, que podem ser causados por osteopenia femoral proximal decorrente do estresse do efeito de proteção. 2 Muitas das hastes modernas foram desenvolvidas para promover o encaixe proximal e com revestimento poroso de hidroxiapatita, que é mais compatível com o denso osso femoral proximal do paciente. Ao utilizar designs cônicos e anatômicos, o encaixe distal da haste é reduzido e hastes mais curtas são empregadas, podendo reduzir a perda óssea proximal em até 14%. 3 Além disso, muitos estudos demonstraram que a revolução do design da haste reduziu a subsidência da haste, a dor na coxa e o afrouxamento. 4 5 No entanto, nenhuma pesquisa comparou a progressão da integração óssea e a perda óssea proximal entre hastes cônicas de cunha dupla (Accolade II, Stryker, Portage, MI, EUA) e hastes anatômicas (ABGII, Stryker). Portanto, o presente estudo compara as diferenças de preenchimento femoral proximal entre esses designs de haste usando radiografias pós-operatórias imediatas e perda óssea femoral proximal utilizando radiografias de acompanhamento. Os resultados proporcionarão uma melhor escolha de modelo de haste, diminuirão as complicações precoces e aumentarão a satisfação com a artroplastia total do quadril.

## Materiais e Métodos

## Delineamento Experimental

Este é um estudo descritivo de coorte retrospectiva de radiografias pós-operatórias imediatas e de acompanhamento após a cirurgia de artroplastia total de quadril realizada entre 2017 e 2019. O Comitê de Ética do hospital (identificação: 62133) aprovou o protocolo de pesquisa e dispensou a exigência de consentimento informado para este estudo. Todos os dados e identificadores coletados dos pacientes foram anonimizados por completo.

## Amostras

## Critérios de Inclusão

Com a permissão do departamento de radiologia de Rajavithi, pacientes com idade entre 18 e 80 anos submetidos à artroplastia total primária do quadril usando os dois tipos de hastes entre 2017 e 2019 e tinham radiografias de acompanhamento por até um ano foram incluídos neste estudo.

## Critérios de Exclusão

Foram excluídos pacientes menores de 18 anos, submetidos à artroplastia de quadril de revisão, com displasia de quadril prévia, qualquer complicação pós-operatória e radiografias de acompanhamento de menos de 1 ano.

## Coleta e Medida de Dados

Obtivemos radiografias pós-operatórias imediatas e de acompanhamento do departamento de radiologia de pacientes submetidos à artroplastia total do quadril, abrangendo um período de até um ano. A análise dos dados foi baseada no método das taxas de preenchimento do canal femoral e realizada pelo cirurgião ortopedista responsável pela reconstrução de quadril e joelho em adultos. 6 Os dados consistiam na medida do diâmetro femoral proximal e do diâmetro da haste em incidência anteroposterior em três níveis: trocânter menor (TM), 2 cm proximal ao TM e 6 cm distal ao TM ( Fig. 1 ).

**Fig. 1:** Medida das taxas de preenchimento do canal no trocânter menor (TM), 2 cm proximal ao TM e 6 cm distal ao TM.

A radiografia de acompanhamento foi analisada quanto a alterações na densidade óssea femoral proximal usando o método de densitometria ideal, 7 empregando o Image J (domínio público), um programa de análise de imagem óptica digital para Windows, que mediu alterações ósseas nas zonas de fixação de Gruen 1, 4 e 7 8 ( Fig. 2 ).

**Fig. 2:** Medida da densidade óssea femoral proximal pelo método de densitometria ideal.

## Análise Estatística

Estatísticas descritivas (número, percentual, média, mediana, desvio-padrão e valores mínimo e máximo) foram utilizadas para descrever as características das amostras. O teste do Qui-quadrado comparou os dados categóricos. O teste *t* pareado foi utilizado para comparar dados independentes, como tipos de haste e fêmur. Além disso, o teste *t* foi empregado para comparação dos dados dependentes, como as radiografias pós-operatórias. O nível de significância foi definido como valores de *p* < 0,05. Todas as análises estatísticas foram realizadas no programa IBM SPSS Statistics for Windows (IBM Corp., Armonk, NY, EUA), versão 20.0.

## Resultados

## Dados Demográficos

Uma amostra total de 92 pacientes (22 homens e 70 mulheres) foi incluída neste estudo. A média de idade foi de 53,86 ± 13,00 anos. Foram 34 pacientes a receber hastes anatômicas (ABGII) e 58 pacientes hastes cônicas em cunha dupla (Accolade II).

Ao comparar as taxas de preenchimento do canal entre as duas hastes, a taxa de preenchimento do canal com a haste cônica em cunha dupla foi significativamente maior do que no grupo que recebeu hastes anatômicas em todos os três níveis de medição ( *p* < 0,001, *p* < 0,001 e *p* = 0,013), como mostra a Tabela 1 .

**Tabela 1:** Comparação das taxas de preenchimento do canal entre os dois tipos de hastes

Nível	Haste anatômica (n = 34)	Haste cônica de cunha dupla (n = 58)	Diferença (intervalo de confiança de 95%)	Valor de *p*
Trocânter menor	81,56	88,13	−6,57 (−9,74 a −3,39)	< 0,001*
2 cm abaixo do trocânter menor	85,98	93,49	−7,51 (−10,07 a −4,95)	< 0,001*
6 cm abaixo do trocânter menor	78,58	85,64	−7,06 (−12,56 a −1,56)	0,013*

Nota: *Estatisticamente significativo (valor de *p* <0,05).

A Tabela 2 apresenta as alterações na densidade óssea femoral com cada tipo de haste nas zonas de Gruen 1, 4 e 7. As duas hastes levaram a uma perda óssea do fêmur proximal entre o valor basal e todos os períodos avaliados.

1. Alterações na densidade óssea proximal com a haste anatômica
Aos 6 meses pós-operatórios, houve diferenças significativas na perda óssea femoral nas zonas 1, 4 e 7 ( *p* = 0,024, *p* < 0,001 e *p* = 0,006, respectivamente); a maior perda óssea femoral foi observada na zona 4 (5,74%). Em 1 ano após a cirurgia, houve diferença significativa na perda óssea femoral nas zonas 1, 4 e 7 ( *p* < 0,001); a maior perda óssea femoral foi encontrada na zona 7 (20,65%). Aos 2 anos após a cirurgia, diferenças significativas na perda óssea femoral foram observadas nas zonas 1 ( *p* < 0,001), 4 ( *p* = 0,004) e 7 ( *p* < 0,001); a maior perda óssea femoral foi observada na zona 1 (34,48%).
2. Alterações na densidade óssea proximal com a haste cônica em cunha dupla
Aos 6 meses pós-operatórios, houve uma diferença significativa na perda óssea femoral nas zonas 1, 4 e 7 ( *p* < 0,001); a maior perda óssea femoral foi observada na zona 1 (8,28%). Com 1 ano de cirurgia, houve diferença significativa na perda óssea femoral nas zonas 1, 4 e 7 ( *p* < 0,001); a maior perda óssea femoral foi observada na zona 1 (12,48%). Aos 2 anos após a cirurgia, foi encontrada uma diferença significativa na perda óssea femoral nas zonas 1, 4 e 7 ( *p* < 0,001); a maior perda óssea femoral foi observada na zona 1 (22,37%).

**Tabela 2:** Alterações na densidade óssea femoral com cada tipo de haste nas zonas de Gruen 1, 4 e 7

Fêmur	Haste anatômica (n = 34)				Haste cônica de cunha dupla (n = 58)			
	Pós-operatório (basal)	6 meses	1 ano	2 anos	Pós-operatório (basal)	6 meses	1 ano	2 anos
**Zona 1** DOF (%; média ± DP) Alteração (%)	135,59 ± 12,20	131,15 ± 10,53 −4,44	117,35 ± 14,19 −13,79	84,74 ± 7,29 −34,48	130,33 ± 13,30	122,05 ± 15,14 −8,28	109,57 ± 12,09 −12,48	91,63 ± 9,06 −22,37
Valor de *p*		0,024*	< 0,001*	< 0,001*		< 0,001*	< 0,001*	< 0,001*
**Zona 4** DOF (%; média ± DP) Alteração (%)	164,03 ± 16,41	158,29 ± 17,11 −5,74	145,74 ± 16,92 −12,56	135,39 ± 12,74 −13,65	152,86 ± 29,44	148,14 ± 24,19 −4,72	138,64 ± 18,49 −9,50	129,77 ± 11,34 −16,23
Valor de *p*		0,001*	< 0,001*	0,004*		0,098	< 0,001*	< 0,001*
**Zona 7** DOF (%; média ± DP) Alteração (%)	157,85 ± 13,84	152,71 ± 11,31 −5,15	132,06 ± 12,33 −20,65	107,83 ± 19,79 −29,57	152,29 ± 13,11	144,29 ± 14,85 −8,00	135,12 ± 14,89 −9,17	126,87 ± 10,59 −11,13
Valor de *p*		0,006	< 0,001*	< 0,001*		< 0,001*	< 0,001*	< 0,001*

**Abreviaturas:** DOF, densidade óssea femoral; DP, desvio padrão.

Nota: *Estatisticamente significativo (valor de *p* <0,05).

Comparando a perda óssea femoral proximal entre os dois designs, a haste cônica em cunha dupla demonstrou perda óssea femoral proximal significativamente menor na zona de Gruen 7 ( Fig. 3 ). No entanto, não houve diferença significativa na perda óssea femoral proximal nas zonas 1 e 4, como mostrado nas Figs. 4 5 e na Tabela 3 .

**Fig. 3:** Comparação das alterações da densidade óssea femoral proximal na zona de Gruen 7 de ambas hastes (A, haste anatômica; B, haste cônica em cunha dupla).

**Fig. 4:** Comparação das alterações da densidade óssea femoral proximal na zona de Gruen 1 de ambas hastes (A, haste anatômica; B, haste cônica em cunha dupla).

**Fig. 5:** Comparação das alterações da densidade óssea femoral proximal na zona de Gruen 4 de ambas hastes (A, haste anatômica; B, haste cônica em cunha dupla).

**Tabela 3:** Comparação das alterações na densidade óssea femoral em cada zona com os dois tipos de hastes

Densidade óssea femoral	Tipo de haste		Valor de *p*
	Haste anatômica (n = 34)	Haste cônica de cunha dupla (n = 58)	
**Zona 1**			
Pós-operatório (basal)	135,59 ± 12,20	130,33 ± 13,30	0,062
6 meses	131,15 ± 10,53	122,05 ± 15,14	0,003*
1 ano	117,35 ± 14,19	109,57 ± 12,09	0,006*
2 anos	84,74 ± 7,29	91,63 ± 9,06	0,004*
Alteração (1 ano de pós-operatório)	−18,24 ± 20,48	−20,76 ± 9,36	0,501
**Zona 4**			
Pós-operatório (basal)	164,03 ± 16,41	152,86 ± 29,44	0,022*
6 meses	158,29 ± 17,11	148,14 ± 24,19	0,021*
1 ano	145,74 ± 16,92	138,64 ± 18,49	0,070
2 anos	135,39 ± 12,74	129,77 ± 11,34	0,096
Alteração (1 ano de pós-operatório)	−18,29 ± 14,79	−14,22 ± 24,22	0,320
**Zona 7**			
Pós-operatório (basal)	157,85 ± 13,84	152,29 ± 13,11	0,058
6 meses	152,71 ± 11,31	144,29 ± 14,85	0,005*
1 ano	132,06 ± 12,33	135,12 ± 14,89	0,314
2 anos	107,83 ± 19,79	126,87 ± 10,59	< 0,001*
Alteração (1 ano de pós-operatório)	−25,79 ± 15,85	−17,17 ± 13,23	0,010*

Nota: *Estatisticamente significativo (valor de *p* <0,05).

A Tabela 3 mostra que houve diferenças significativas nas alterações da densidade óssea femoral entre ambas as hastes apenas na zona 7 ( *p* = 0,01).

## Discussão

A artroplastia total do quadril não cimentada é um procedimento popular, principalmente em pacientes mais jovens, 9 com bom desfecho em longo prazo. No entanto, há relatos de osteopenia femoral proximal e afrouxamento asséptico precoce com os primeiros designs 4 devido ao efeito de proteção contra estresse e ao micromovimento proximal da haste. Posteriormente, a haste foi refinada pela melhora da superfície e do material de revestimento, assim como redução da rigidez do material e a variação do comprimento da haste femoral, todos melhoram muito a sobrevida e reduzem as chances de complicações no procedimento. 10

Nosso estudo descobriu que a taxa de preenchimento do canal com a haste cônica em cunha dupla foi significativamente maior do que no grupo com haste anatômica em todos os níveis (TM, 2 cm acima do TM e 6 cm abaixo do TM). Vale ressaltar que o maior preenchimento do canal femoral e a maior proporção de preenchimento do canal observados em nosso estudo podem aumentar o risco de falha da osteointegração, como sugerido por Cooper et al, cujo estudo relata que um aumento no preenchimento médio e distal e no índice de alargamento do canal é o fator de risco mais importante para falha da osteointegração. 11

De acordo com nosso estudo sobre alterações na densidade óssea periprotética, a densidade mineral óssea pós-operatória imediata no lado operado deve ser usada como valor basal para exclusão de perda óssea devido ao procedimento cirúrgico. 12 Apesar desse método, nosso estudo observou perda de densidade óssea com as duas hastes a partir do valor basal, que atribuímos à proteção contra estresse na área. Este achado é consistente com o estudo de Venesmaa et al., 13 que relatou uma diminuição geral em todas as regiões de interesse até 6 meses, particularmente na região de Calcar, e apenas pequenas alterações após esse período. Entretanto, nosso estudo também observou diminuição da densidade óssea em até 1 ano após a cirurgia. Descobrimos que na zona 7, a perda de densidade óssea femoral foi significativamente maior em pacientes com haste anatômica do que naqueles com haste em cunha cônica dupla (−25% *versus* −17%, *p* = 0,010). No entanto, nenhuma perda significativa de densidade óssea femoral foi observada nas zonas 1 e 4.

O ponto forte deste estudo é sua condução por um único cirurgião em um único centro, o que minimizou fatores de confusão da técnica cirúrgica e do cuidado pós-operatório do paciente. Este estudo, porém, tem limitações. Primeiro, sua natureza retrospectiva, o que limitou a coleta de dados sobre todos os fatores que podem ter influenciado a perda óssea em nossa população. Além disso, o tamanho da amostra foi relativamente pequeno, o que nos impediu de realizar uma análise significativa de subgrupos para investigar o impacto de vários fatores na perda óssea. Em segundo lugar, o período de acompanhamento foi curto (12 meses), mas acreditamos que foi adequado, uma vez que a perda de densidade óssea periprotética foi mais pronunciada no primeiro ano pós-operatório, com alterações mínimas posteriormente. Este achado é consistente com estudos anteriores que destacaram o processo inicial de remodelação óssea periprotética nos primeiros 12 meses de pós-operatório. 14

## Conclusão

A haste em cunha cônica dupla teve uma taxa de preenchimento do canal significativamente maior do que a haste anatômica em todos os níveis, com menor perda de densidade óssea femoral identificada na radiografia pós-operatória de acompanhamento da zona 7. No entanto, nas zonas 1 e 4, nenhuma diferença significativa foi observada na perda de densidade óssea femoral.
